# Hypoxia-induced PVT1 promotes lung cancer chemoresistance to cisplatin by autophagy via PVT1/miR-140-3p/ATG5 axis

**DOI:** 10.1038/s41420-022-00886-w

**Published:** 2022-03-07

**Authors:** Jiying Wang, Zhiyi Dong, Zhaoying Sheng, Yong Cai

**Affiliations:** 1 Department of Oncology, Shanghai Pulmonary Hospital, Tongji University School of Medicine, 200433 Shanghai, China; 2 Department of Traditional Chinese Medicine, Shanghai Pulmonary Hospital, Tongji University School of Medicine, 200433 Shanghai, China; 3 Department of Radiation Oncology, Shanghai Pulmonary Hospital, Tongji University School of Medicine, 200433 Shanghai, China

**Keywords:** Lung cancer, Molecular biology

## Abstract

Lung cancer is one of the most common and lethal malignant tumors and the cases increased rapidly. Elevated chemoresistance during chemotherapy resistance remains a challenge. Hypoxia is one of the components that lead to chemoresistance. PVT1 participates in various tumor drug resistance and is associated with hypoxia conditions. The present study aimed to analyze the regulatory relationship of hypoxia and PVT1 and the mechanism of PVT1 in the hypoxia-induced chemoresistance process of lung cancer. The expression of PVT1 in lung cancer and adjacent tissues, and cell lines were analyzed using the TCGA database and qPCR. The regulatory relationship between hypoxia and PVT1 was validated and analyzed with qPCR, luciferase reporter system, and CHIP-qPCR. The role of PVT1 in chemoresistance ability induced by hypoxia was analyzed with CCK-8 assay and flow cytometry. The roles of PVT1, hypoxia, and chemoresistance were also analyzed with LC3-GFP transfection, WB, and IHC. Finally, the results were further validated in xenograft models. PVT1 is highly expressed in lung cancer and cell lines, and the expression of PVT1 is regulated by HIF-1α, and the luciferase reporter assay and CHIP-qPCR analysis indicated that HIF-1α could bind to the promoter region of PVT1 and regulate PVT1 expression. PVT1 participated in hypoxia-induced chemoresistance and induced higher viability and lower apoptosis rate by the autophagy signaling pathway via PVT1/miR-140-3p/ATG5 axis. All the findings were validated in the xenograft models. In conclusion, these results suggest that the expression of PVT1 is regulated by HIF-1α and participates in hypoxia-induced chemoresistance.

## Introduction

Lung cancer is one of the most common types of malignant tumors. According to the cancer epidemiological statistics data, almost 228,820 newly diagnosed cases and 135,720 cancer deaths appeared worldwide in 2020 [1]. The origin of lung cancer is associated with oncogene mutations such as EGFR, KRAS, PTEN, and DDR2 [2]. Cisplatin (DDP) is one of the first-line chemotherapy drugs and is widely applied in the treatment of lung cancer [3]. However, the long-term treatment of DDP always leads to a series of side effects, and the therapeutic effect decreases with the development of chemoresistance [4]. Therefore, it is worth better understanding the chemoresistance mechanism of lung cancer and developing more efficient therapies for lung cancer.

The tumor microenvironment (TME) is the environment around the tumor that combines various kinds of cells and plays a vital role in tumor progression [5]. The rapid growth of the tumor results in hypoxia condition in the internal tissues [6]. The hypoxic environment leads to cancer cells proliferation, migration, chemoresistance, and immune escape by regulating hypoxia-inducible factor (HIF)-1α-related genes and signaling pathways [7]. Hypoxia could promote the chemoresistance ability of colon cancer and lung cancer to 5-Fu and adriamycin by upregulating Pgp expression [8–11], while in gastric cancer and melanoma cancer, hypoxia promotes chemoresistance by activating the P53 and nuclear factor-κB signaling pathways [12, 13]. Furthermore, the autophagy signaling pathway plays a significant role in regulating cellular chemoresistance to cisplatin in lung cancer [14, 15]. The unfolded protein response could enhance the autophagy signaling pathway and promote the enhancement of chemoresistance [16].

Long noncoding RNA (lncRNA) is a kind of noncoding RNA that is>200 nt and participates in regulating various cellular progresses, such as proliferation, migration, and chemoresistance [17]. The former researches demonstrated that PVT1 promoted cancer progression by promoting proliferation, migration, or inhibiting apoptosis in colon cancer, breast cancer, and pancreatic cancer [18–21]. The chemoresistance ability was also regulated by PVT1 regulating autophagy signaling pathway, Wnt signaling pathway, and PI3K/AKT signaling pathway in cervical, osteosarcoma, or pancreatic cancer [22–24]. PVT1 is highly expressed in various types of cancer, the expression level of PVT1 is commonly associated with the hypoxia process [25]. Plenty of researches showed that PVT1 promoted hypoxia progression by various regulatory mechanisms. However, whether hypoxia regulates PVT1 expression is still unclear. Furthermore, whether PVT1 participates in the hypoxia-induced chemoresistance process is also unclear.

In this study, we validated the regulatory relation of HIF-1α and PVT1 by chromatin immunoprecipitation (ChIP)–quantitative real-time polymerase chain reaction (qPCR) and luciferase reporter assay. The role of PVT1 in hypoxia-induced chemoresistance was evaluated and validated with cell counting kit-8 (CCK-8) assay and flow cytometry. Finally, our results revealed a novel mechanism of hypoxia-induced chemoresistance and suggested a promising treatment target for lung cancer.

## Results

### PVT1 is highly expressed in lung cancer tissue and is up-expressed in hypoxia condition

**Fig. 1:**
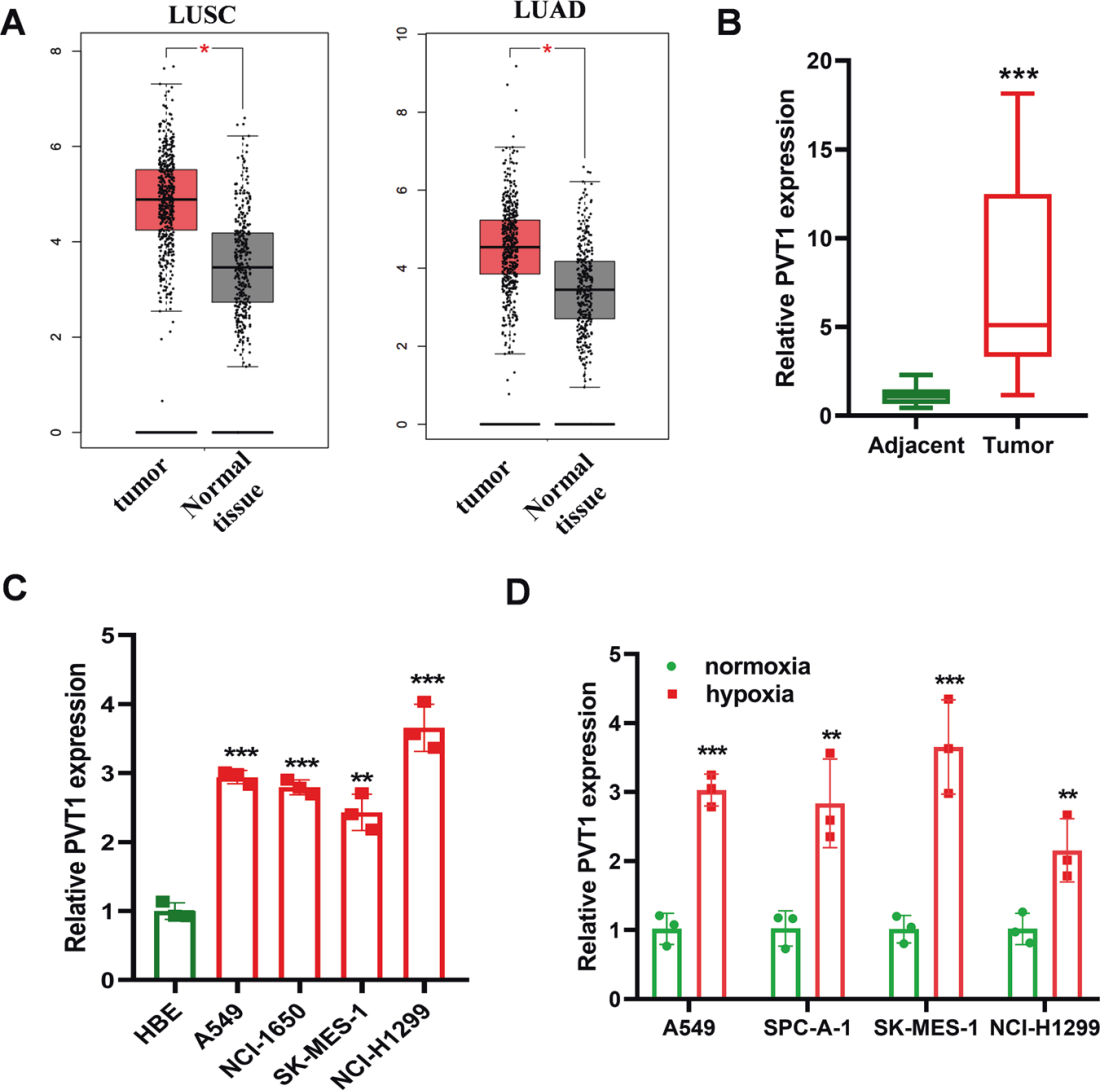
PVT1 is highly expressed in lung cancer tissue and up-expressed in hypoxia conditions. **A** The expression level of PVT1 in LUAD and LUSC tissues and the adjacent tissues in TCGA through GEPIA2 analysis; **B** qPCR analysis of PVT1 expression between lung cancer tissue and adjacent tissues; *n* = 15; **C** qPCR analysis of PVT1 expression between lung cancer cell lines and normal cell; **D** qPCR analysis of PVT1 expression in lung cancer cell lines under hypoxia or normoxia conditions. *P < 0.05, **P < 0.01, ***P < 0.001.

To assess the role of PVT1 in lung cancer progression, the expression level of PVT1 between LUAD, LUSC tissues, and normal tissues from The Cancer Genome Atlas was explored using GEPIA 2 online tool [26]. As shown in Fig. 1A, PVT1 is highly expressed in LUAD and LUSC tissue compared to normal tissue. We then validated the expression pattern with qPCR. As shown in Fig. 1B, C, PVT1 is highly expressed in lung cancer tissue and the cancer cell lines. Hypoxia is a syndrome of solid tumors and promotes cancer progression by regulating various genes expression. To find the regulatory effect of hypoxia on PVT1 expression, qPCR was employed. As shown in Fig. 1D, the expression level of PVT1 was significantly elevated under hypoxia conditions and we selected A549 and SK-MES-1 cells for further research. Then, we treated the cells with CoCl_2_, a chemical inducer of HIF-1, and we also found a significant elevation of PVT1 expression in A549 and SK-MES-1 cells (Supplementary Fig. 1A), and knockdown of HIF-1α expression via shRNAs also demonstrated that HIF-1α regulated PVT1 expression under hypoxia conditions, but barely affected PVT1 expression under normoxia (Supplementary Fig. 1B). All the results suggest that PVT1 is regulated by HIF-1α under hypoxia conditions.

### Hypoxia regulates PVT1 expression via HIF-1α

**Fig. 2:**
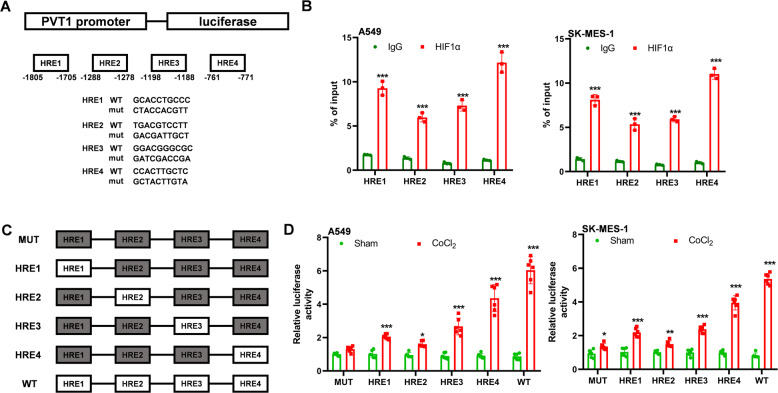
Hypoxia regulates PVT1 expression via HIF-1α. **A** The HRE locations and mutation sequence of PVT1 promoter; **B** CHIP-qPCR analysis of the enrichment of HRE sequences in PVT1 promoter; **C** construction of the reporter sequence of PVT1 promoter, the gray box means the mutation form of the HRE and the white box means the WT form of HRE; **D** luciferase analysis of the regulatory effects of HIF-1α on PVT1 transcription; *P < 0.05, **P  < 0.01, ***P < 0.001.

We then analyzed which factor induces PVT1 expression in lung cancer. The promoter sequence (~2 kb) of PVT1 was analyzed with a promoter analysis tool (JASPAR). Four HIF-1α response elements (HRE) were found in the promoter region (Fig. 2A). We validated the four HREs in A549 and SK-MES-1 cells with ChIP-qPCR under hypoxia conditions, as shown in Fig. 2B, HIF-1α could bind to the HRE sites of PVT1 promoter. To determine whether and how HIF-1α regulates PVT1 expression through HREs in the promoter regions, six luciferase reporter vectors were constructed and named as WT, HRE1, HRE2, HRE3, HRE4, and MUT. The plasmids were transfected into A549 or SK-MES-1 cells and the luciferase activity was measured. As expected, CoCl_2_ significantly increased the luciferase activity of cells transfected with WT vectors compared with the MUT vectors. And the HRE4 in the promoter was the main region that HIF-1α bound to and promoted PVT1 expression. These results suggested that hypoxia transcriptionally regulated PVT1 expression by HIF-1α through directly binding with HREs on its promoter.

### PVT1 promotes the chemoresistance ability of A549 and SK-MES-1 cells via autophagy under hypoxia condition

**Fig. 3:**
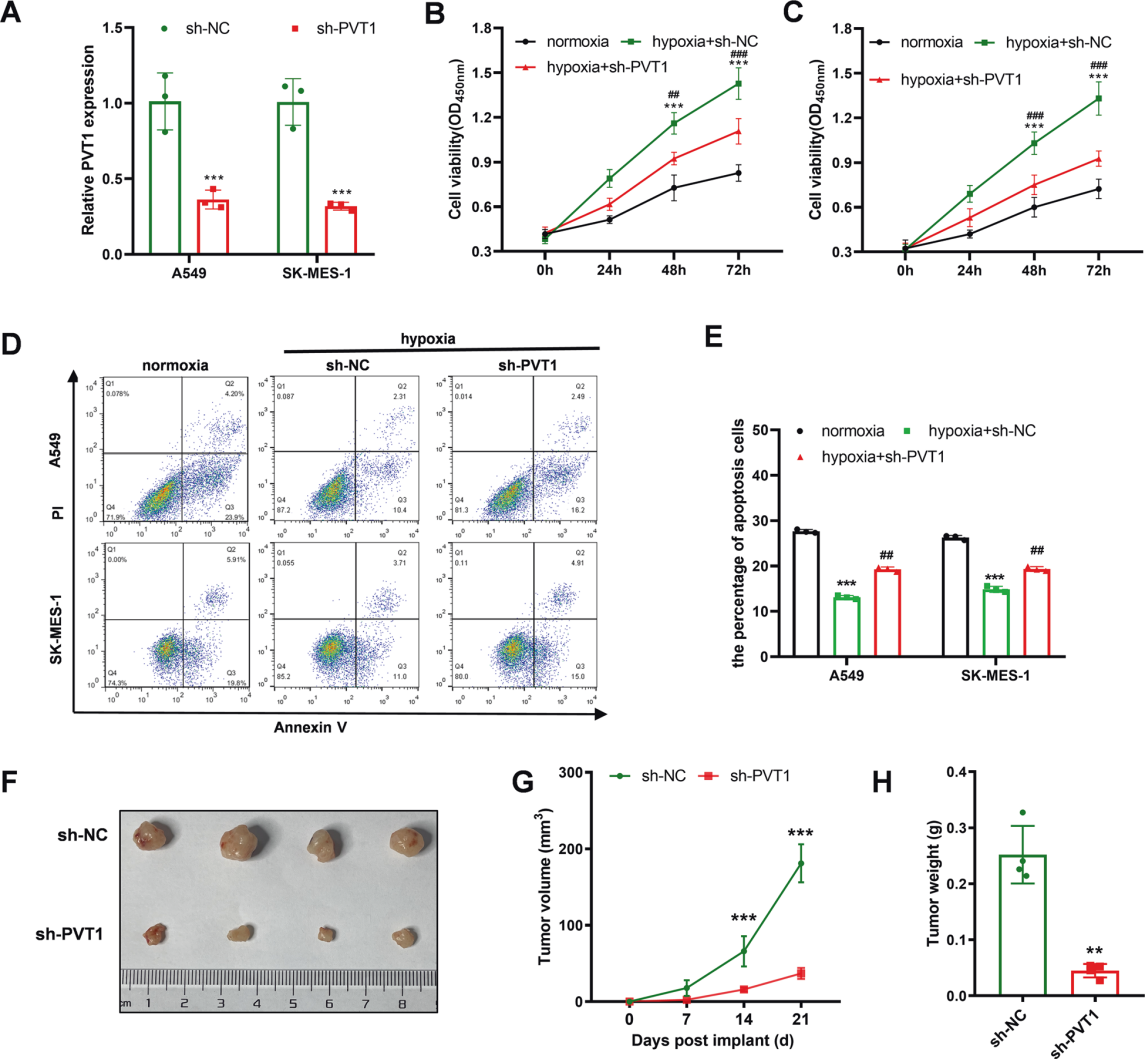
PVT1 promotes the chemoresistance ability of A549 and SK-MES-1 cells under hypoxia conditions. **A** qPCR validation of the knockdown efficiency of sh-PVT1 in A549 and SK-MES-1 cells; **B**, **C** CCK-8 analysis of the chemoresistance to cisplatin of A549 and SK-MES-1 cells under normoxia and hypoxia conditions and hypoxia+sh-PVT1 treatment, and the cells were treated with 50 μM cisplatin; **D**, **E** flow cytometry analysis of the apoptosis rates of A549 and SK-MES-1 cells under normoxia and hypoxia conditions and hypoxia+sh-PVT1 treatment, and the cells were treated with 50 μM cisplatin for 48 h; **F**–**H** the volume of the tumors was measured every 7 days, and the final volume and weight of the tumors of each group were analyzed at 21 days. Asterisk (*) indicates the comparison of normoxia vs. hypoxia, and hash (#) indicates the comparison of hypoxia vs. hypoxia+sh-PVT1. *P < 0.05, **P < 0.01, ***P < 0.001, ^##^*P* < 0.01, ^###^*P* < 0.001.

PVT1 is involved in various cancer progressions, such as proliferation, migration, or chemoresistance [27]. It remains unclear whether PVT1 promotes the chemoresistance ability of lung cancer under hypoxia conditions. Here, we constructed the sh-PVT1 vector and successfully knocked down PVT1 in A549 and SK-MES-1 (Fig. 3A). The role of PVT1 in the chemoresistance of lung cancer cells under hypoxia or normoxia conditions was analyzed with CCK-8 assay. As shown in Supplementary Fig. 2, hypoxia conditions could elevate the chemoresistance ability compared with normoxia conditions. Knocking down PVT1 expression would inhibit chemoresistance ability in normoxia or hypoxia conditions, and the inhibitory effect seems more significant under hypoxia conditions. Whether PVT1 participates in the hypoxia-induced chemoresistance remains unclear. The following CCK-8 analysis showed that hypoxia promoted A549 and SK-MS-1 cells’ chemical resistance ability to cisplatin and promoted cell viability and the elevated chemoresistance and viability were reversed with knocking down PVT1 expression (Fig. 3B, C). The apoptosis cell rates with cisplatin treatment also indicated that the reduction of cell apoptosis rates of A549 and SK-MES-1 cells were reversed with PVT1 knockdown (Fig. 3D, E). The A549 cells transfected with sh-PVT1 or sh-NC plasmid were seeded into the right flank of the mice, and 50 μM cisplatin was injected into the intraperitoneal every three days. Consistent with the cellular results, the tumor size, volume, and weight of the sh-PVT1 A549 cell group were significantly lower than the control group (Fig. 3F–H). All the results demonstrated that the highly expressed PVT1 affected the sensitivity of lung cancer to cisplatin in vitro and in vivo.

**Fig. 4:**
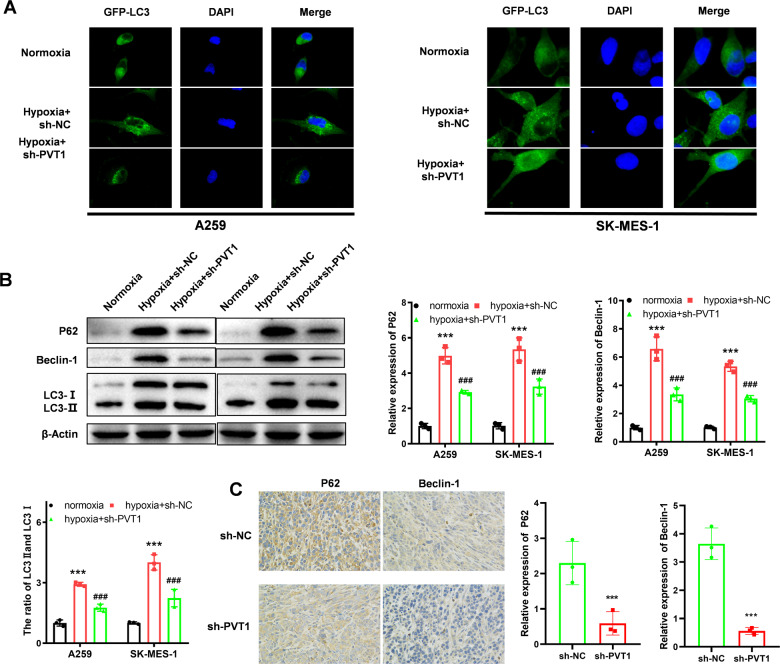
Hypoxia enhances autophagy signaling pathway activity via PVT1. **A** GFP-LC3 analysis of autophagy signaling pathway activity of each group; **B** WB analysis of the autophagy signaling pathway activity of each group; **C** IHC analysis of the autophagy signaling pathway activity (P62, Beclin1, and LC3 I/II) of the xenograft of each group. Asterisk (*) indicates the comparison of normoxia vs. hypoxia, and hash (#) indicates the comparison of hypoxia vs. hypoxia+sh-PVT1. ***P < 0.001, ^###^*P* < 0.001.

To assess the role of hypoxia and PVT1 in regulating autophagy signaling pathways, LC3-GFP fluorescence analysis, WB, and IHC analysis were employed. Upon activation of the autophagy signaling pathway, the LC3 originally distributed in the cytoplasm was recruited to the outer and inner membranes and punctate aggregation occurred. As shown in Fig. 4A, the autophagy activity of A549 or SK-MES-1 cells was elevated under hypoxia conditions, and the elevation was reversed with transfection of sh-PVT1. The quantification of aggregation was shown in Supplementary Fig. 3A. Consistent with the results, the WB analysis also proved hypoxia elevated the activity of the autophagy signaling pathway, and knocking down PVT1 expression reversed the autophagy signaling pathway activity (Fig. 4B). We validated the regulatory relationship in the xenograft tumors with IHC staining, and the results also demonstrated that knocking down PVT1 expression could inhibit the activity of the autophagy signaling pathway (Fig. 4C).

### PVT1 regulates ATG5 expression via sponging miR-140-3p

**Fig. 5:**
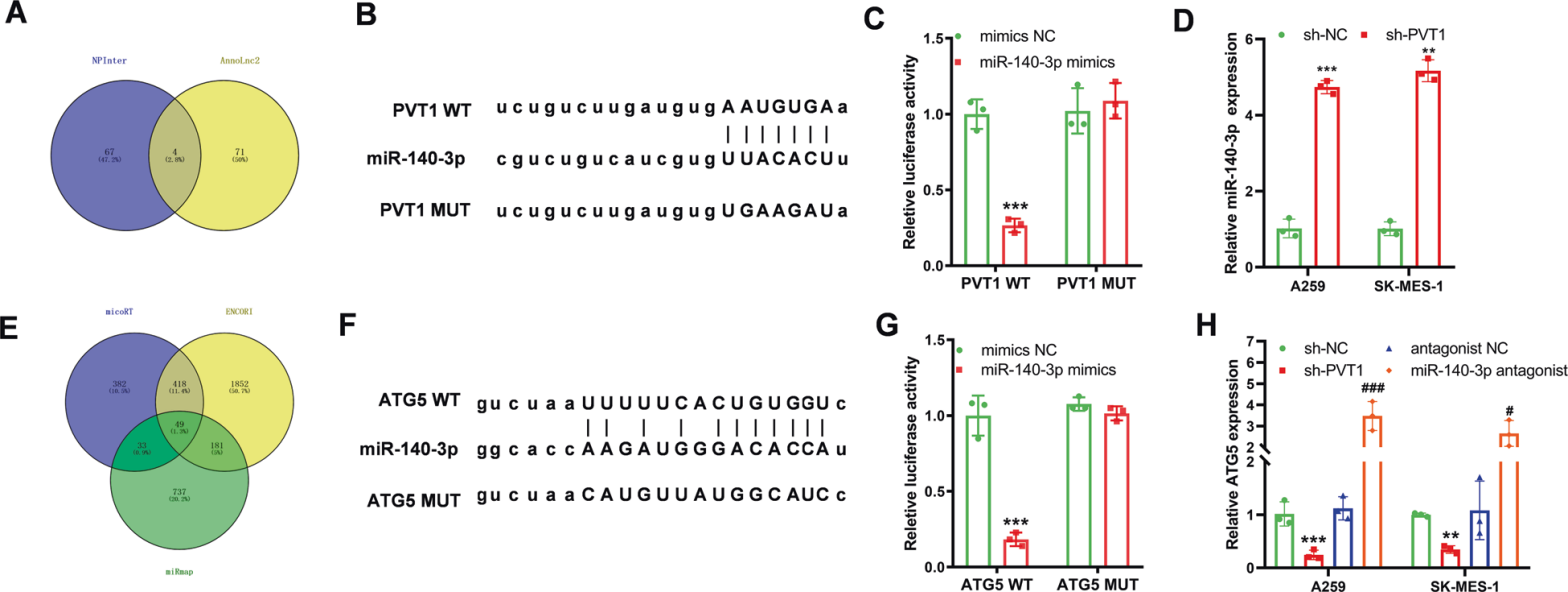
PVT1 regulates autophagy through the miR-140-3p/ATG5 axis. **A** Venny analysis of the targeted miRNAs of PVT1; **B** the binding sites of PVT1 and miR-140-3p were predicted by the NPInter and Annlog2 database; **C** dual-luciferase reporter assay confirmed PVT1 and miR-140-3p binding activity in A549 cells; **D** qPCR was performed to detect the expression level of miR-140-3p in response to sh-PVT1 in A549 cells; **E** Venny analysis of the targeted mRNAs of miR-140-3p; **F** the binding sites of PVT1 and miR-140-3p were predicted by the micoRT, ENCORI, and miRmap database; **G** dual-luciferase reporter assay confirmed miR-140-3p and ATG5 mRNA binding activity in A549 cells; **H** qPCR was performed to detect the expression level of ATG5 mRNA in response to sh-PVT1 and miR-140-3p antagonist in A549 cells. Asterisk (*) indicates the comparison of mimics NC vs. miR-140-3p mimics or sh-NC vs. sh-PVT1, and hash (#) indicates the comparison of antagonist NC vs. miR-140-3p antagonist. **P < 0.01, ***P < 0.001, ^#^*P* < 0.05, ^###^*P* < 0.001.

After identification of the role of PVT1 in promoting lung cancer cells’ chemoresistance ability via activating the autophagy signaling pathway. We subsequently set out to analyze the mechanism of how PVT1 regulates the autophagy signaling pathway. AnnoLnc2 (http://annolnc.gao-lab.org/) and RNAInter (http://www.rnainter.org/) databases were employed to analyze the miRNAs binding to PVT1 [28, 29], and the results were shown by the Venn diagram in Fig. 5A. Among the 5 miRNAs, the miR-140-3p shows the highest score in the AnnoLnc2 database, and the literature mining also showed that miR-140-3p inhibited the autophagy singling pathway in gastric cancer [30]. The same screening methods were employed in miR-140-3p targeted genes. As shown in Fig. 5D, 45 genes were found as the targeted genes. Finally, we set ATG5 as the targeted gene of miR-140-3p. The former research showed that miR-140-3p bound to ATG5 and inhibited the autophagy signaling pathway in Huh7.5.1 cells [31]. The following estimation of the binding site also showed that PVT1 could bind to miR-140-3p and miR-140-3p might bind to the 3’ UTR of ATG5 mRNA (Fig. 5B, F). The luciferase reporter assay also demonstrated that PVT1 could bind to miR-140-3p, and miR-140-3p could bind to ATG5 mRNA in A549 cells (Fig. 5C, G). Knockdown of PVT1 expression significantly increases the expression level of miR140-3p and decreases the expression of ATG5, while knockdown of miR-140-3p with miR-140-3p antagonist elevates the expression of ATG5 in A549 and AK-MES-1 cells (Fig. 5D, H). Thus, we speculated that PVT1 might regulate the autophagy signaling pathway via the miR-140-3p/ATG5 axis.

### PVT1 regulates autophagy through the miR-140-3p/ATG5 axis

**Fig. 6:**
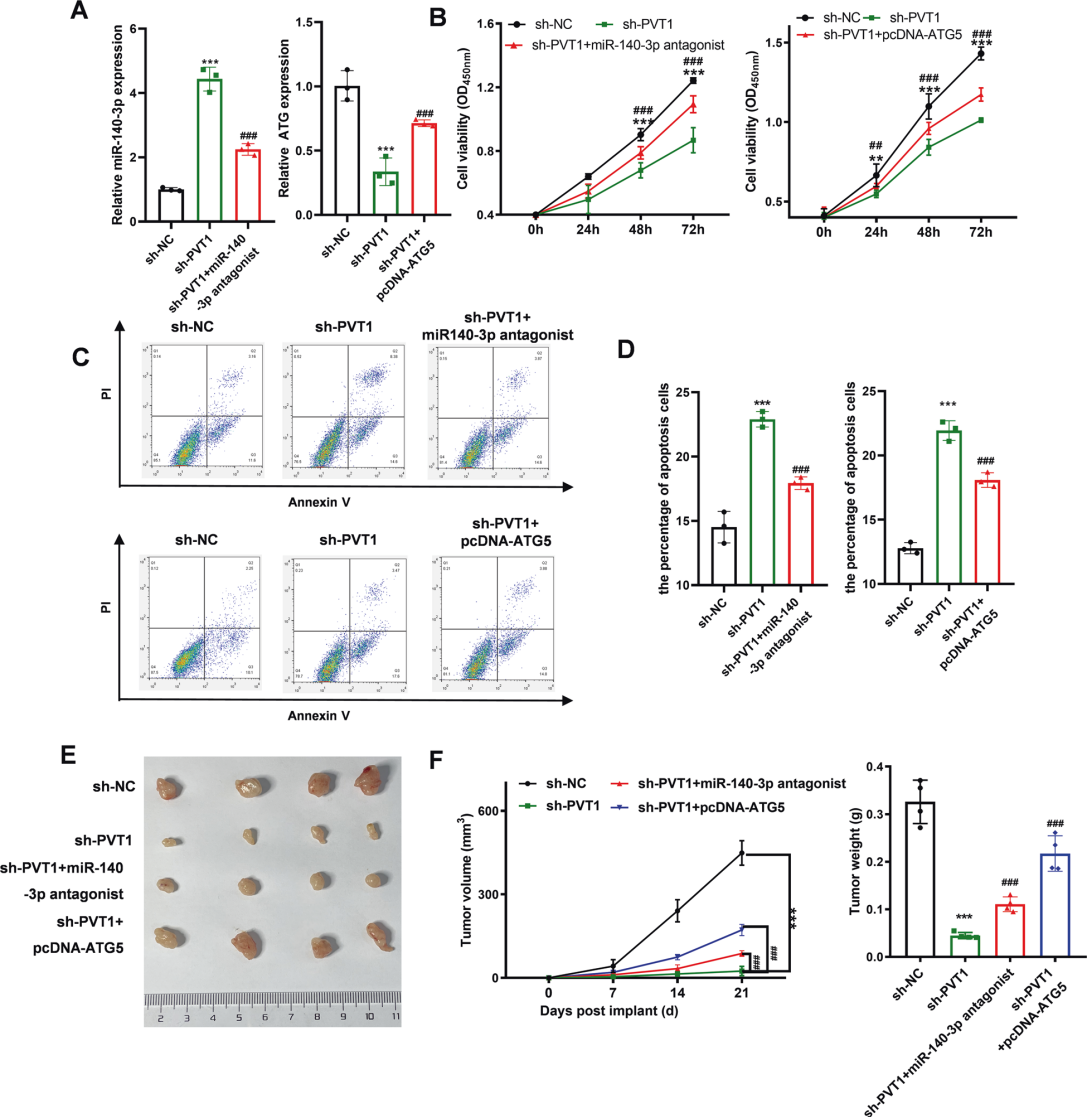
PVT1 regulates autophagy through the miR-140-3p/ATG5 axis. **A** qPCR was used to validate the knockdown efficiency of miR-140-3p or overexpression efficiency of ATG5 in A549 cells; **B** CCK-8 was used to analyze the chemoresistance of A549 cells under hypoxia conditions with sh-PVT1, miR-140-3p antagonist, and pcDNA-ATG5 treatment; **C**, **D** flow cytometry was used to analyze the apoptosis rates of A549 cells under hypoxia conditions after sh-PVT1, miR-140-3p, and pcDNA-ATG5 treatment; **E**, **F** the volume of the tumors of each group was measured every 7 days, and the weight of the tumors of each group was analyzed at 21 days. Asterisk (*) indicates the comparison of sh-NC *vs*. sh-PVT1, and hash (#) indicates the comparison of sh-PVT1 vs. sh-PVT1 + miR-140-3p antagonist or sh-PVT1 + pcDNA-ATG5. **P < 0.01, ***P < 0.001, ^##^*P* < 0.01, ^###^*P* < 0.001.

The former analysis showed that PVT1 could regulate ATG5 expression via miR-140-3p. Rescue experiments were employed to investigate whether PVT1 regulated cell chemoresistance and autophagy by miR-140-3p and ATG5. The expression level of miR-140-3p and ATG5 altered with PVT1 knockdown, and the alteration was reversed with transfection of miR-140-3p antagonist or pcDNA-ATG5 plasmid (Fig. 6A). The chemoresistance ability of A549 was analyzed with CCK-8 and flow cytometry analyses. The CCK-8 analysis showed that the decreased viability of A549 cells under hypoxia conditions induced by PVT1 knocking down was reversed with transfection of miR-140-3p antagonist or pcDNA-ATG5 plasmid (Fig. 6B). The promotion of cell apoptosis rates of A549 cells induced by knockdown of PVT1 under hypoxia conditions was reversed with transfection of miR-140-3p antagonist or pcDNA-ATG5 plasmid (Fig. 6C, D). Consistent with the cellular results, the xenograft results also indicated that the inhibition of chemoresistance induced by knockdown of PVT1 was reversed by downregulating miR-140-3p or overexpression of ATG5 (Fig. 6E, F).

**Fig. 7:**
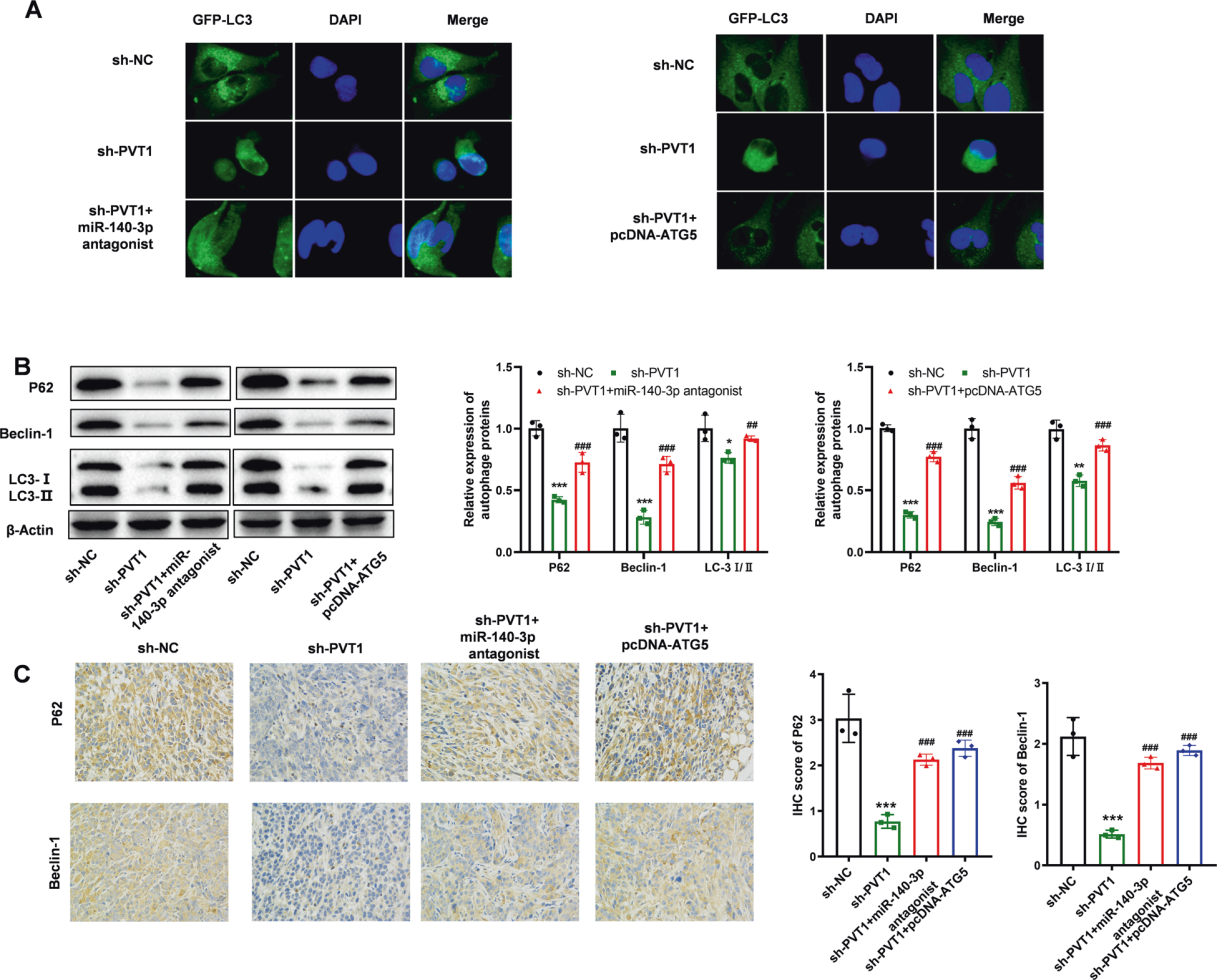
PVT1 regulates autophagy through the miR-140-3p/ATG5 axis. **A** LC3 analysis of autophagy signaling pathway activity of each group; **B** WB analysis of the autophagy signaling pathway activity of each group; **C** IHC analysis of the autophagy signaling pathway activity of the xenograft of each group. Asterisk (*) indicates the comparison of sh-NC vs. sh-PVT1, and hash (#) indicates the comparison of sh-PVT1 vs. sh-PVT1 + miR-140-3p antagonist or sh-PVT1 + pcDNA-ATG5. *P < 0.05, **P < 0.01, ***P < 0.001, ^##^*P* < 0.01, ^###^*P* < 0.001.

The mechanism analysis also demonstrated that miR-140-3p antagonist or over-expression of ATG5 could reverse the decreased autophagy signaling pathway induced by knocking down PVT1 (Fig. 7). As shown in Fig. 7A and Supplementary Fig. 3B, the LC3-GFP was enriched in the cytoplasm and granular aggregation in the hypoxia group, while knocking down PVT1 expression caused the less granular aggregation. However, the transfection of miR-140-3p antagonist or pcDNA-ATG5 reversed the decrease of granular aggregation in the cell cytoplasm. The following protein expression of P62, Beclin-1, and the ratio of LC3 I/II also revealed that the decreased autophagy activity induced by knocking down PVT1 was reversed by transfection of miR-140-3p antagonist or pcDNA-ATG5. The IHC analysis of xenograft tumors also revealed consistent results (Fig. 7C). All the results demonstrated that PVT1 could promote A549 cell chemoresistance ability by promoting the autophagy signaling pathway via miR-140-3p/ATG5 axis.

## Discussion

Drug resistance has been considered a serious challenge for cancer therapies [32]. The main mechanisms of chemoresistance can be grouped as the insufficiency of pharmacokinetic properties, intrinsic factors of tumor cells, and external conditions of tumor cells in the tumor microenvironment [33]. With the rapid proliferation of the tumors, the vessels could not deliver enough oxygen to the core of the tumor and finally induced hypoxia [34]. Hypoxia induces various proteins and lncRNA expression with HIF-1α or HIF-2α and finally promotes tumor progression and chemoresistance [35]. The hypoxia-induced lncRNAs such as UPK1A, UPK1A-AS1, and circAKT3 participate in the genesis of chemoresistance [36, 37]. Therefore, finding the hypoxia-related lncRNAs and analyzing the mechanism might be a way to find the therapeutic targets in the treatment of lung cancer.

Here, we demonstrated that PVT1 was highly expressed in LUAD and LUSC tissues, and the expression of PVT1 was regulated by hypoxia and HIF-1α. PVT1 participated in hypoxia-induced chemoresistance of lung cancer cells by promoting the autophagy signaling pathway via the PVT1/miR-140-3p/ATG5 axis. Together, our results present a new mechanism that PVT1 is a hypoxia-related lncRNA and participates in the hypoxia-induced chemoresistance process by regulating the autophagy signaling pathway via PVT1/miR-140-3p/ATG5 axis in lung cancer.

Recent research has indicated that PVT1 was frequently upregulated in various tumors and promoted tumor progression by promoting the proliferation, migration and some other signaling pathways [27, 38]. The former researches indicated that PVT1 participated in hypoxia-induced gene expression via stabilizing HIF-1α or as a ceRNA for miR-199a-5p [39, 40]. The research also suggested that hypoxia enhanced the expression of PVT1 in lung cancer cells. However, whether and how hypoxia regulates PVT1 expression remain unknown. Our results demonstrated that PVT1 was highly expressed in lung cancer tissue and cell lines. And hypoxia conditions could promote PVT1 expression via HIF-1α binding to the HREs of the PVT1 promoter region. The estimation score of HIF-1α and PVT1 promoter and the following ChIP-qPCR analysis also showed that HRE4 might be the most important binding site. Our result validated the regulatory relationship of hypoxia and PVT1 and verified that HIF-1α directly promoted PVT1 expression by binding to the promoter region of PVT1. HIF-2α is also the hypoxia-induced transcription factor and participates in the various hypoxia-induced process and the role of HIF-2α in regulating PVT1 or other targets genes remains for further analysis [41].

Hypoxia is one of the characteristics of the tumor microenvironment and enhances the chemoresistance ability of the tumors. The former research also indicated that PVT1 could promote the chemoresistant ability to gemcitabine and cisplatin by regulating Wnt, autophagy, or PI3K/AKT signaling pathway in osteosarcoma or pancreatic cancer [22, 24, 42]. Thus, we assume that hypoxia-induced chemoresistance might be at least partly caused by PVT1. Consistent with the assumption, the enhanced chemoresistance ability induced by hypoxia was significantly reversed by transfection with sh-PVT1. The autophagy signaling pathway is an adaptive response to stresses, such as starvation, hypoxia, and chemotherapy drugs [43]. It was also shown that PVT1 promoted pancreatic cancer and glioma progression via the autophagy signaling pathway [22, 44, 45]. In our results, we demonstrated that the activation of the autophagy signaling pathway was positively associated with hypoxia, and hypoxia-induced PVT1 expression and enhanced autophagy signaling pathway activity was saved with PVT1 knockdown. Briefly, we reveal that the enhanced ability of hypoxia-induced chemoresistance was mainly induced by the PVT1 regulated autophagy signaling pathway.

LncRNAs regulate the expression of the targeted gene at transcription and post-transcription levels [46]. The post-transcription regulation mainly relies on forming the duplex of miRNAs and lncRNA and inducing the miRNA degradation. We focused on the potential miRNAs that might connect PVT1 and the autophagy signaling pathway activity. We screened out the targeted miRNAs and mRNAs through multiple sources and validated the interaction and regulation relationship with luciferase analysis and qPCR (Fig. 5). Finally, we found that PVT1 could regulate the autophagy signaling pathway via the PVT1/miR-140-3p/ATG5 axis. miR-140 shows decreased expression in basal cell carcinoma, breast cancer, and lung cancer and plays a suppression role in tumor proliferation and metastasis ability [47–51]. And the elevated expression of miR-140-3p significantly inhibits the chemoresistance ability to 5-Fu, cisplatin in gastric cancer, osteosarcoma, and lung cancer via regulating the autophagy or Wnt signaling pathway [52–54]. We analyzed the miR-140-3p targeted mRNAs in multiple databases and finally found candidate genes. Among the 40 genes, we finally found that miR-140-3p could bind to the 3’UTR of ATG5 and the following research also revealed that miR-140-3p degrade the mRNA of ATG5. Consistently, the latest research also demonstrated that the expression of ATG5 was also regulated by miR-140-3p in gastric cancer progression [55]. ATG5 was the key regulatory gene of the autophagy signaling pathway, and our results suggested that the hypoxia/PVT1/miR-140-3p signaling axis regulated the chemoresistance ability via regulating ATG5 expression in lung cancer. Finally, our research demonstrated that hypoxia-induced PVT1 could enhance the chemoresistance ability via the PVT1/miR-140-3p/ATG5 axis.

In total, we found that PVT1 is a kind of hypoxia-associated lncRNA, and promotes chemoresistance by autophagy signaling pathway via PVT/miR-140-3p/ATG5 under hypoxia conditions. Our results provide a better understanding of hypoxia-induced chemoresistance and the hypoxia/PVT1/miR-140-3p/ATG5/autophagy signaling axis may be a novel treatment target for lung cancer.

## Materials and methods

### Clinical samples

A total of 15 lung cancer tissues and adjacent tissues were collected from the patients that received surgical operations at Shanghai Pulmonary Hospital, Tongji University School of Medicine. The tissues were stored in a −80 °C fridge for further RNA extraction. All the patients have been informed of the contents before the surgery, and the research was carried out following the Ethics Committee of Shanghai Pulmonary Hospital, Tongji University School of Medicine.

### Cell culture and treatments

The human lung cancer cell lines (HBE, A549, SK-MES-1, NCI-H1299, and NCI-H1650) were obtained from American Type Culture Collection (Manassas, VA, USA) and cultured in DMEM medium that combines 10% fetal bovine serum (FBS, Gibico, USA) and 1% P/S. The cells were cultured under optimal conditions (5% CO_2_ and 37 °C). For the hypoxia treatment, the cells were cultured in the tri-gas incubator (Thermo Fisher, USA), and the N_2_ and CO_2_ gas were added to the incubator and construct a culture environment with 1% O_2_, 5% CO_2,_ and 94% N_2_.

### qPCR analysis

Total RNA was isolated from the cells using Trizol (Thermo Fisher, USA). After purification, RNA was subjected to synthesize cDNA using PrimeScript™ 1st Strand cDNA Synthesis Kit (TAKARA, JP). qPCR was performed with IQ^TM^ SYBR green supermix (BIO-RAD, JP). The glyceraldehyde-3-phosphate dehydrogenase (GAPDH) and endogenous small nuclear RNA U6 were employed as the housekeeping genes for lncRNA/mRNA or miRNA. The 2^−ΔΔCt^ method was employed to calculate the relative expression of PVT1, miR-140-3p, and ATG5. The primers were listed as follows: PVT1 (Forward, 5′-CATCCGGCGCTCAGCT-3′; Reverse, 5′-TCATGATGGCTGTATGTGCCA-3′), miR-140-3p (Forward, 5′-CAGTGCTGTACCACAGGGTAGA-3′; Reverse, 5′-TATCCTTGTTCACGACTCCTTCAC-3′) ATG5 (Forward, 5′-GACAAAGATGTGCTTCGAGATGTG-3′ and Reverse, 5′-GTAGCTCAGATG CTCGCTCAG-3′); U6 (Forward, 5′-ATTGGAACGATACAGAGAAGATT-3′; Reverse, 5′-GGAACGCTTCACGAATTTG-3′); GAPDH (Forward, 5′-TGTTCGTCATGGGTGTGAAC-3′; Reverse, 5′-ATGGCATGGACTGTGGTCAT-3′).

### Cell transfection

Transfection of RNA oligonucleotides and plasmids were performed with lipo2000 (Thermo Fisher, USA) as the protocol. Briefly, a final concentration of 20 nM RNA or plasmids was mixed with lipo2000 and added to the cell culture medium, and the medium was replaced at 8-hours later. After 48 h, the expression of target genes was measured with qPCR.

### CCK-8 assay

During the analysis, the cells were treated with 50 μM cisplatin. Cell viability was analyzed by the CCK-8 according to the manufacturer’s protocol. Briefly, cells were seeded to the 96-well plates with a concentration of 3 × 10^3^ cells per well. The freshly prepared CCK-8 detection solution (10 µl) was added to the well and incubated at 37 °C for 2 h. The OD value was detected with a microplate reader at 450 nm. Each analysis was repeated at least three times.

### Cell apoptosis analysis with flow cytometry

Cells were treated with 50 μM cisplatin for 48 h before the analysis. Cells were collected and washed with PBS (phosphate-buffered saline). After centrifugation, cells were resuspended in 100 μl binding buffer, then 5 μl of annexin V-FITC and 5 μl of propidium iodide (PI) were added into the cell suspension, and incubated for 15 min at room temperature. After washing with PBS, cell apoptosis was analyzed by flow cytometry.

### Western blotting (WB)

Cells were lysed with RIPA buffer that contained 1% proteinase and phosphatases inhibitor cocktail (Beyond time, China). The protein samples were quantified with BCA (beyond time, China), and 30 μg of them were separated by 10% SDS-PAGE and transferred to NC membranes (Sangon Biotech, China). The NC membranes were blocked with block buffer and incubated with primary antibody overnight at 4 °C. After the overnight incubation, the membrane was washed and incubated with the second antibody for 2 h at room temperature. The expression of targeted proteins was analyzed using the ECL detection system (Thermo Fisher, USA) and quantified by densitometry using ImageJ software. The antibody and dilution were listed below: LC3-I/II (Abcam, USA, 1:2000), Beclin-1 (Abcam, USA, 1:1000), P62 (Abcam, USA, 1:10000), Actin (Abcam, USA, 1:10000).

### Plasmid construction

The HRE binding sites were analyzed on the jasper website (http://jaspar.genereg.net/). The promoter of PVT1 was cloned from A549 cell cDNA and cloned into a pMIR-reporter vector. Mutation PCR was applied to construct the specific HRE mutation vector and then cloned to the vector. Finally, we constructed the WT (wild type), HRE1 positive, HRE2 positive, HRE3 positive, HRE4 positive, and HRE mutation PVT1 promoter. The sequence of PVT1 and 3’UTR of ATG5 sequence that binds to miR140-3p were cloned to pMIR-reporter, and mutant PCR was applied to construct the mutant sequence of the binding site. ATG5 sequence was amplified from the A549 cDNA and cloned to the pcDNA3.1 plasmid. For the sh-PVT vector, the sequence of AACUCCUCAGCCUCCAAGCTT was cloned to the pLKO.1 vector.

### Luciferase reporter assay

The WT or mutant pMIR-ATG5 or pMIR-PVT1 vectors and miR-140-3p mimics were co-transfected into A549 cells with lipo2000. Forty-eight hours later, the relative luciferase activity of each group was measured with the microplate reader (Thermo, USA). The pMIR-PVT1 promoter vector was transfected to the A549 or SK-MES-1 cells, and then the cells were subgrouped into two groups and named as NC group and CoCl_2_ group. The CoCl_2_ group was treated with 150 M CoCl_2_. After 48 h, the relative luciferase activity was measured with the microplate reader (Thermo, USA).

### ChIP-qPCR analysis

A549 or SK-MES-1 cells were fixed with 1% formaldehyde for 15 min and then quenched with glycine. The cell lysate was treated with sonication to obtain the chromatin fragment of 300-500 bp. HIF-1α antibody and control IgG were employed to immunoprecipitation. Then protein A-Sepharose was added and incubated for 1 h at room temperature. After de-crosslinking, DNA was purified for qRT-PCR. All qRT-PCR reactions were done in triplicates. The primers were shown below: HRE1 (Forward, 5′-ACAACTAGATGCCAACTGCA-3′; Reverse, 5′-GTCTGTTTTGTGCCCGGCTC-3′), HRE2 (Forward, 5′-CCCATTTGTGTGTCCAATT-3′; Reverse, 5′-CAGCTAGGCAATGGGG-3′), HRE3 (Forward, 5′-CTCCCCTTAAGTGCTCAGTA-3′; Reverse, 5′-TGGGATTACAGGCGTGAG-3′), HRE4 (Forward, 5′-AAGATAACCACATCCCACAT-3′; Reverse, 5′-ATGCCCAGTAGTTCCTCT-3′).

### GFP-LC3 fluorescence

pEGFP-LC3 (Addgene, USA) was transiently transfected into A549 or SK-MES-1 cells as previously described [56]. The cells were fixed and the nuclei were stained with DAPI (Invitrogen, USA). Finally, the results were imaged under a fluorescence microscope (Nikon, Japan). The results were photographed with a fluorescence microscope (Echo-labs, USA).

### Xenograft animal model

All the animal experiments were approved by the Institutional Animal Care and Use Committee of Shanghai Pulmonary Hospital, Tongji University School of Medicine. Briefly, 4-week-old, male BALB/c nude mice were obtained from Shanghai Institute of Planned Parenthood Research. A549 cells (2 × 10^6^) were implanted to the right flank of the mice (n = 6 each group). Cisplatin (50 μM) was injected into the intraperitoneal every 3 days. The tumor size was recorded every seven days by calculating the length and the width of the tumor. On day 21, the mice were asphyxiated with carbon dioxide, the tumors were isolated and weighed, and in the end, the tissue was stored for further qPCR or IHC analysis.

### Immunohistochemistry (IHC) staining assay

The tissue sections were deparaffinized, rehydrated using xylene and graded ethanol series, and heated in citrate buffer for antigen retrieval. Then, the sections were washed, blocked, and incubated with normal goat serum. Then, the samples were incubated with a primary anti-P62 (Abcam, USA, 1:500) or anti-Beclin-1 (Abcam, USA, 1:300) antibody at 4 °C overnight and a secondary antibody (Abcam, USA, 1:5000). The sections were stained with diaminobenzidine (DAB) and then counterstained with hematoxylin, dehydrated, and mounted. At last, the section was photographed using a digital microscope camera (Nikon, Japan). The images were qualified through a combination of stain intensity and the percentage of positive cells. Briefly, 0 for no reaction or weak reaction, 1 for intense focal or diffuse weak reaction; 2 for moderate diffuse reaction; and 3 for intense diffuse reaction. The stain intensity score was counted and scored as 1,3,5. And the percentage of positive cells was counted. The final score of IHC was the combination of the stain intensity score and the percentage of positive cells.

### Statistical analysis

Prism 8.0 was applied for the statistical analysis of data. The data of each figure were expressed as mean ± SE. Student’s test (two-tailed) was employed to analyze the significance of two groups. And three or more group comparisons were performed by one-way analysis of variance and Tukey test. *P* < 0.05, 0.01, and 0.001 were considered significantly different.

## Supplementary information

Supplemental Material

Supplementary Figures 1-3

## Data Availability

All data generated or analyzed during this study are included in this article and its Supplementary Information files.
