# Supplementary material for: Hypoxia-induced PVT1 promotes lung cancer chemoresistance to cisplatin by autophagy via PVT1/miR-140-3p/ATG5 axis
Source: Cell Death Discov. 2022 Mar 7;8:104. doi: 10.1038/s41420-022-00886-w (PMC8901807; doi:10.1038/s41420-022-00886-w)
Supplement: Supplementary file 1 — Supplemental Material [file 41420_2022_886_MOESM1_ESM.pptx]

## Slide 1
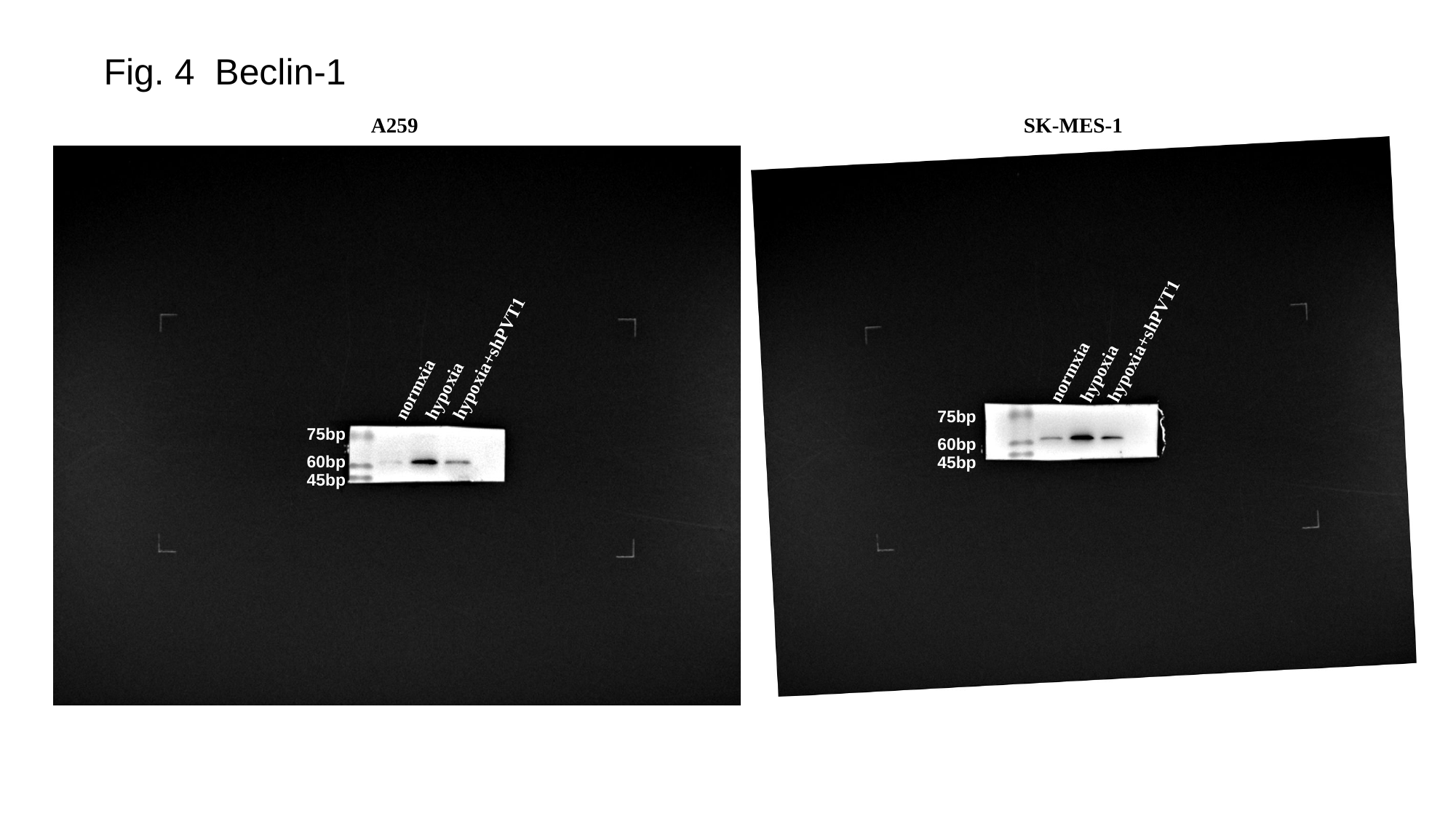

Fig. 4 Beclin-1
A259
SK-MES-1
normxia
hypoxia
hypoxia+shPVT1
normxia
hypoxia
hypoxia+shPVT1
75bp
75bp
60bp
60bp
45bp
45bp

## Slide 2
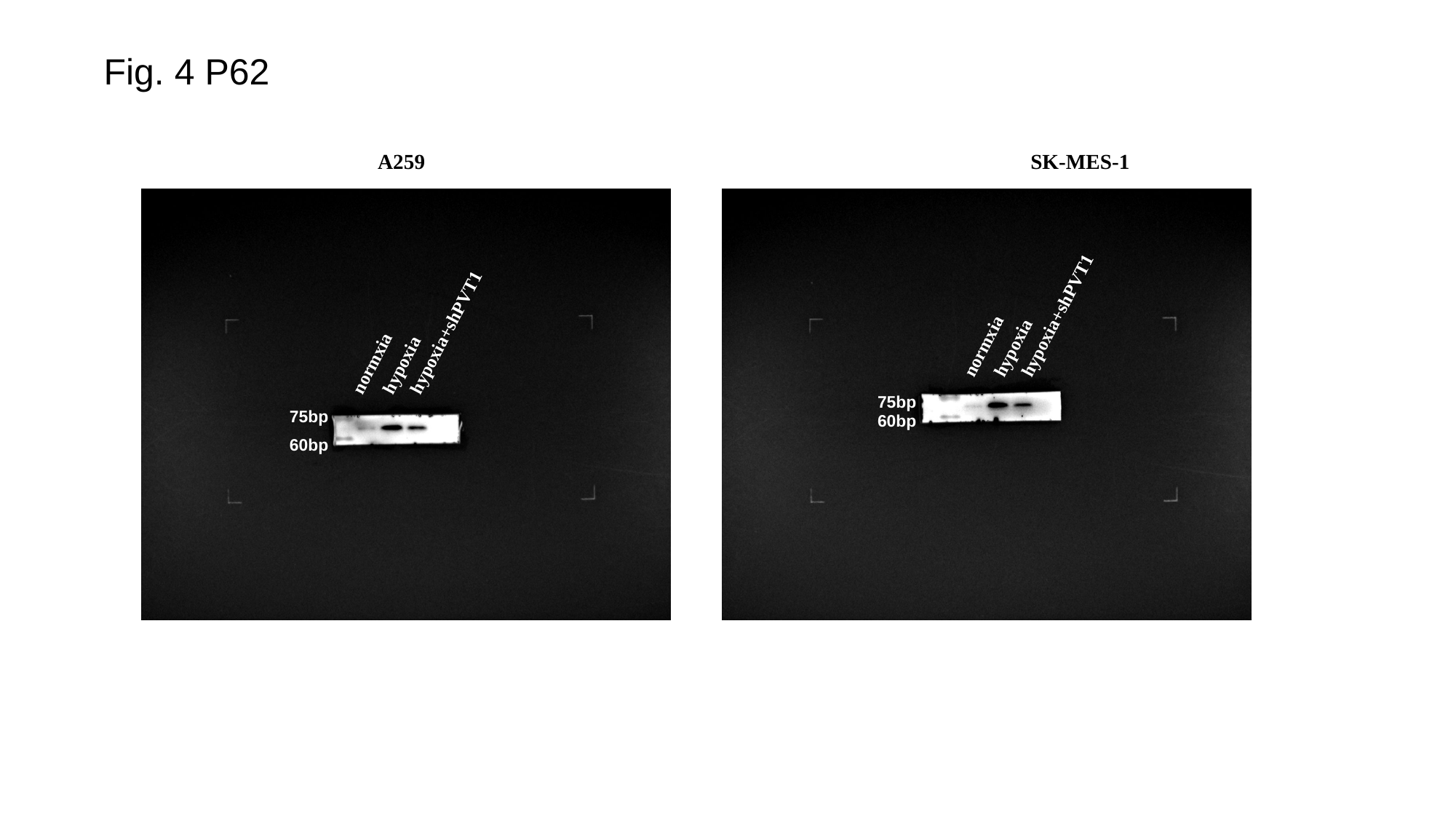

Fig. 4 P62
A259
SK-MES-1
normxia
hypoxia
hypoxia+shPVT1
normxia
hypoxia
hypoxia+shPVT1
75bp
75bp
60bp
60bp

## Slide 3
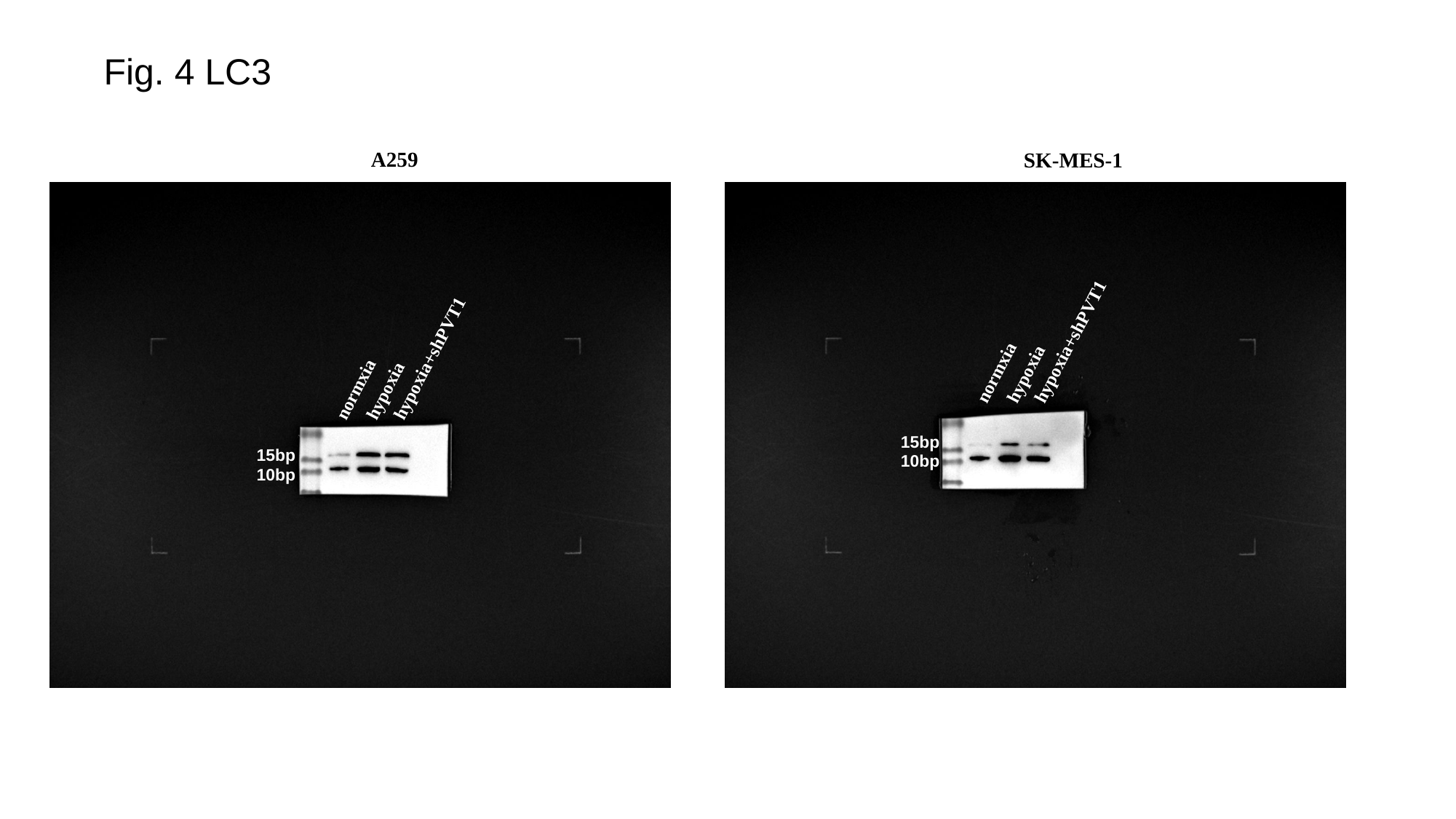

Fig. 4 LC3
A259
SK-MES-1
normxia
hypoxia
hypoxia+shPVT1
normxia
hypoxia
hypoxia+shPVT1
15bp
15bp
10bp
10bp

## Slide 4
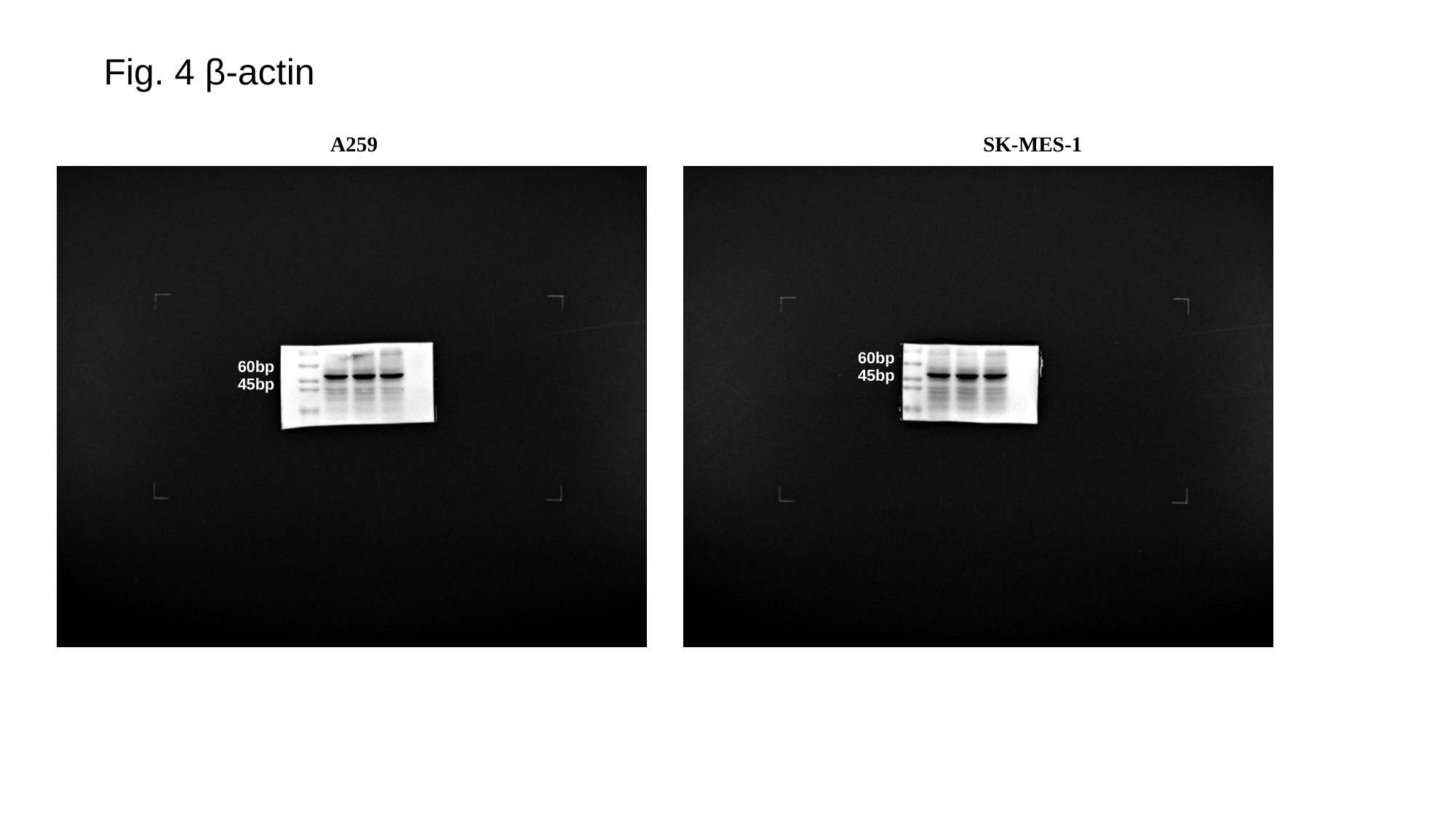

Fig. 4 β-actin
A259
SK-MES-1
60bp
60bp
45bp
45bp

## Slide 5
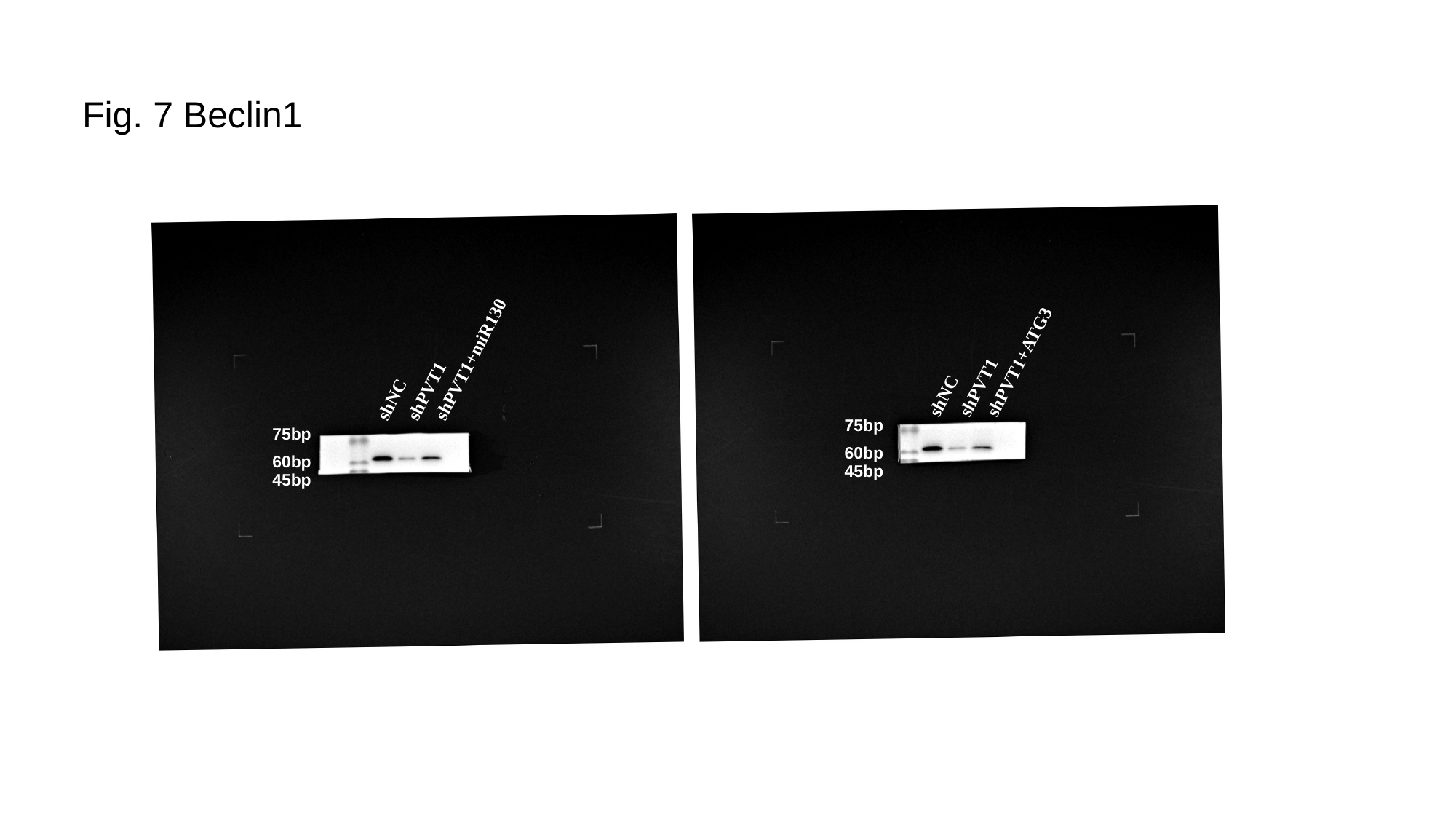

Fig. 7 Beclin1
75bp
60bp
45bp
75bp
60bp
45bp
shNC
shPVT1
shPVT1+ATG3
shNC
shPVT1
shPVT1+miR130

## Slide 6
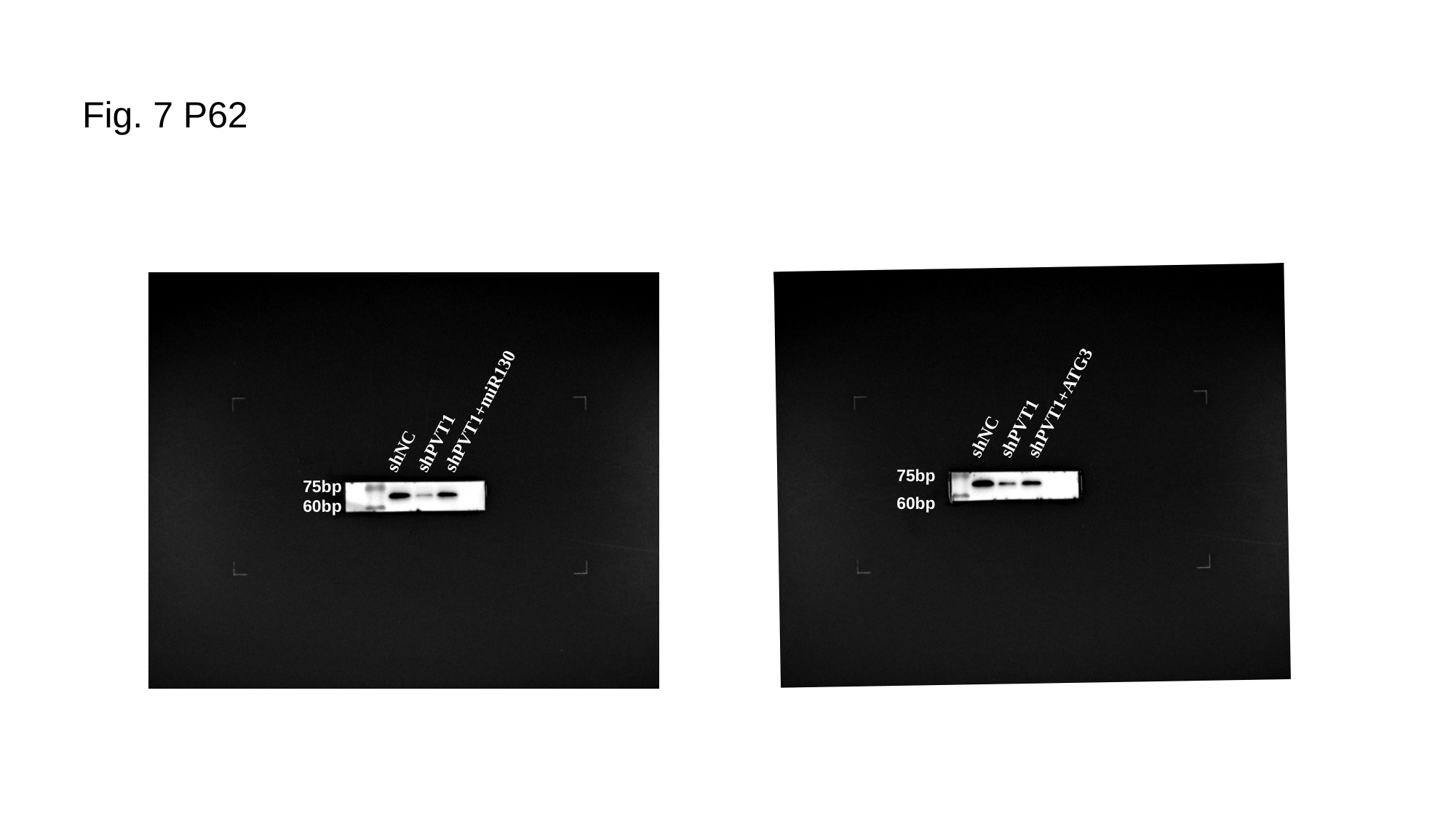

Fig. 7 P62
shNC
shPVT1
shPVT1+ATG3
shNC
shPVT1
shPVT1+miR130
75bp
75bp
60bp
60bp

## Slide 7
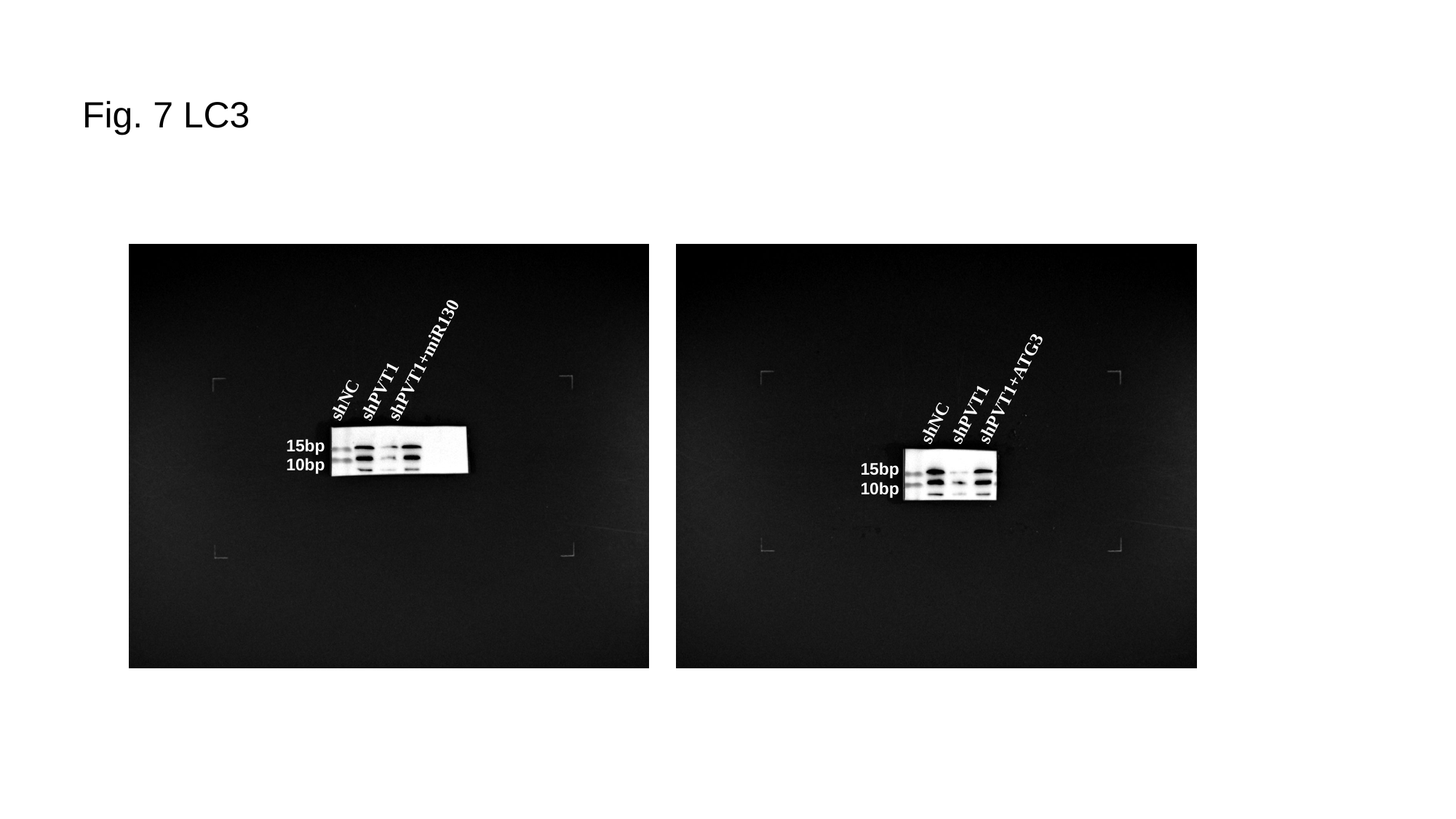

Fig. 7 LC3
shNC
shPVT1
shPVT1+miR130
shNC
shPVT1
shPVT1+ATG3
15bp
10bp
15bp
10bp

## Slide 8
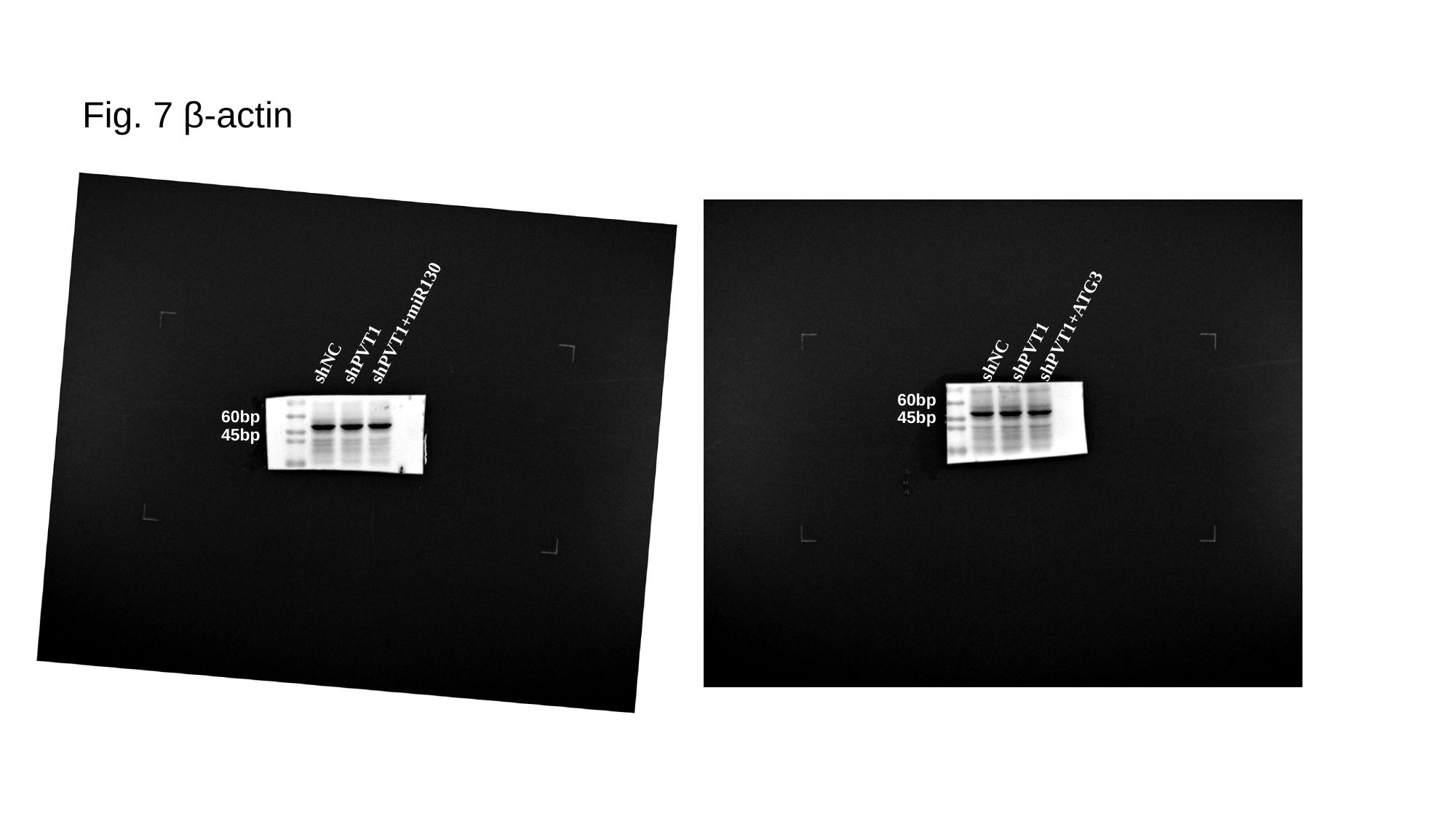

Fig. 7 β-actin
shNC
shPVT1
shPVT1+miR130
shNC
shPVT1
shPVT1+ATG3
60bp
60bp
45bp
45bp
