# Supplementary material for: Hypoxia-induced PVT1 promotes lung cancer chemoresistance to cisplatin by autophagy via PVT1/miR-140-3p/ATG5 axis
Source: Cell Death Discov. 2022 Mar 7;8:104. doi: 10.1038/s41420-022-00886-w (PMC8901807; doi:10.1038/s41420-022-00886-w)
Supplement: Supplementary file 2 — Supplementary Figures 1-3 [file 41420_2022_886_MOESM2_ESM.doc]

**
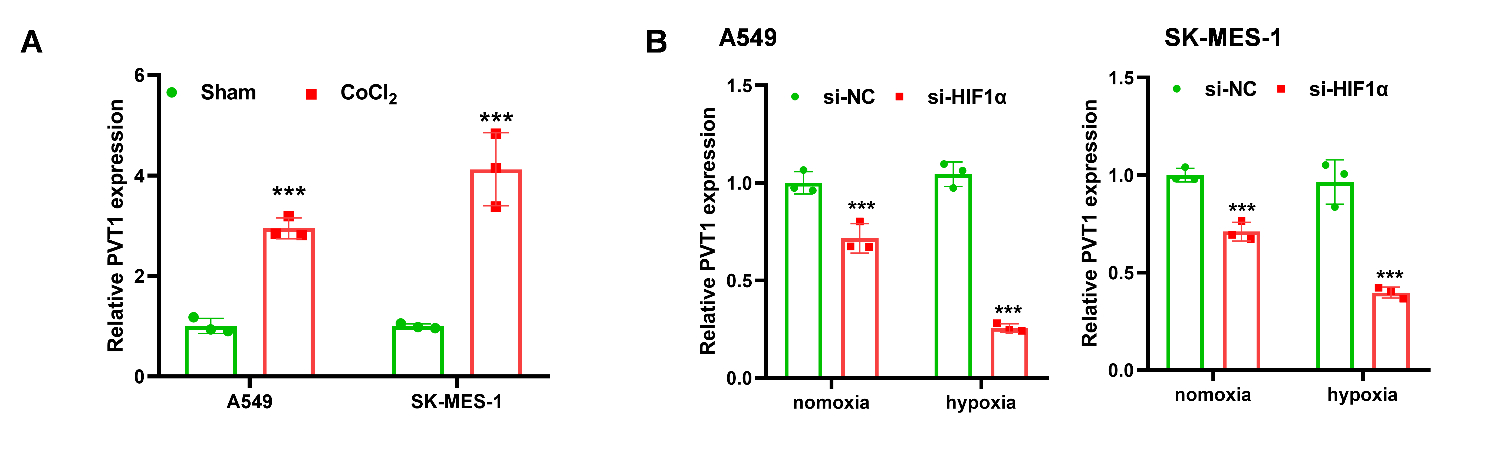
**

**Supplementary Figure 1** HIF-1α regulates PVT1 expression in lung cancer cells. (A) qPCR analysis of the expression of PVT1 of each group; (B) qPCR analysis of the expression of PVT1 of each group. *** *p* < 0.001.

**
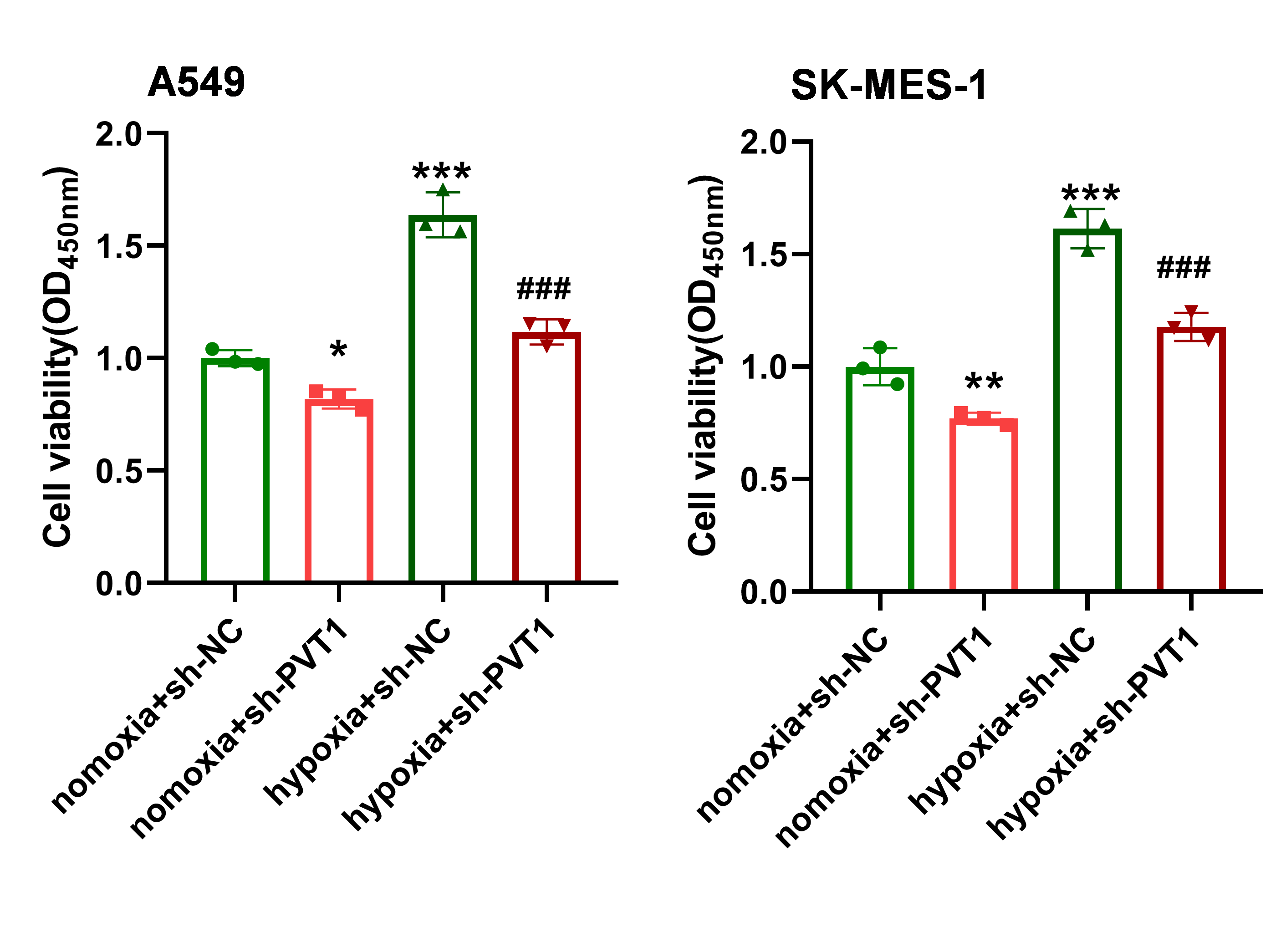
**

**Supplementary Figure 2** CCK-8 analysis of the cell viability. * indicating the comparison of normoxia+sh-NC *vs.* normoxia+sh-PVT1 or hypoxia+sh-NC, and # indicating the comparison of hypoxia+sh-NC *vs.* hypoxia+sh-PVT1. * *p* < 0.05, ** *p* < 0.01, ****p* < 0.001, ### *p* < 0.001.

**
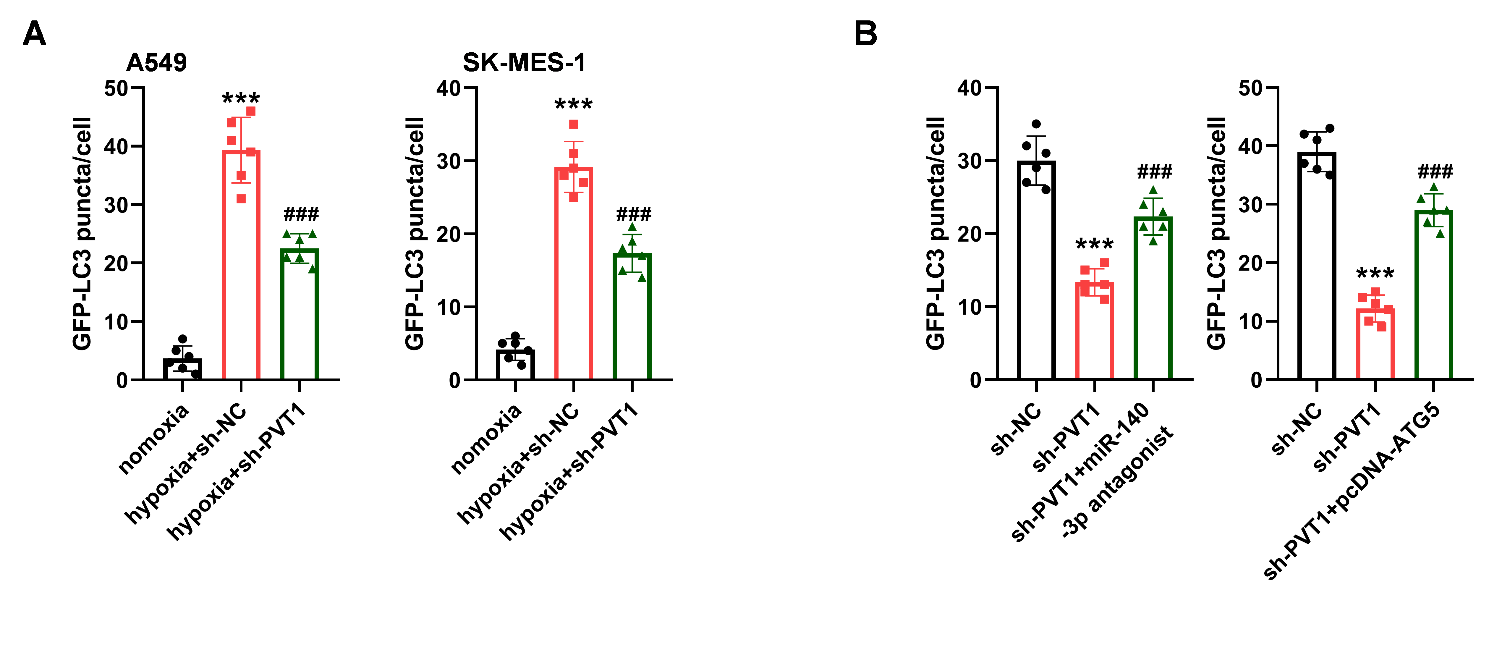
**

**Supplementary Figure 3** Quantification of GFP-LC3 aggregation level of the cell. (A) Quantification of GFP-LC3 aggregation level of each group in Figure 4A, * indicating the comparison of normoxia *vs.* hypoxia, and # indicating the comparison of hypoxia *vs.* hypoxia+sh-PVT1. ** *p* < 0.01, ****p* < 0.001. (B) Quantification of GFP-LC3 aggregation level of each group in Figure 7A, * indicating the comparison of sh-NC *vs.* sh-PVT1, and # indicating the comparison of sh-PVT1 *vs.* sh-PVT1+miR-140-3p antagonist or sh-PVT1+pcDNA-ATG5. ** *p* < 0.01, ****p* < 0.001, ### *p* < 0.001.
